# Multiple‐cumulative probabilities used to cluster and visualize transcriptomes

**DOI:** 10.1002/2211-5463.12327

**Published:** 2017-11-13

**Authors:** Xingang Jia, Yisu Liu, Qiuhong Han, Zuhong Lu

**Affiliations:** ^1^ School of Mathematics Southeast University Nanjing China; ^2^ State Key Laboratory of Bioelectronics School of Biological Science and Medical Engineering Southeast University Nanjing China; ^3^ Linyi No. 1 High School of Shandong Province Linyi China; ^4^ Department of Mathematics Nanjing Forestry University China

**Keywords:** icc‐cluster, multiple‐cumulative probabilities, PCC‐MCP, t‐SNE‐MCP

## Abstract

Analysis of gene expression data by clustering and visualizing played a central role in obtaining biological knowledge. Here, we used Pearson's correlation coefficient of multiple‐cumulative probabilities (PCC‐MCP) of genes to define the similarity of gene expression behaviors. To answer the challenge of the high‐dimensional MCPs, we used icc‐cluster, a clustering algorithm that obtained solutions by iterating clustering centers, with PCC‐MCP to group genes. We then used *t*‐statistic stochastic neighbor embedding (t‐SNE) of KC‐data to generate optimal maps for clusters of MCP (t‐SNE‐MCP‐O maps). From the analysis of several transcriptome data sets, we demonstrated clear advantages for using icc‐cluster with PCC‐MCP over commonly used clustering methods. t‐SNE‐MCP‐O was also shown to give clearly projecting boundaries for clusters of PCC‐MCP, which made the relationships between clusters easy to visualize and understand.

**Abbreviations**
ED‐MCP: Euclidean distance of multiple‐cumulative probabilities
FP of t‐SNE: the first components of t‐SNE projections
icc‐cluster: a clustering algorithm that obtains solutions by iterating clustering centers
MCP: multiple‐cumulative probability
PCA: principal component analysis
PCC‐MCP: Pearson's correlation coefficient of multiple‐cumulative probabilities
PCC: Pearson's correlation coefficient
SOM: self‐organizing map
SP of t‐SNE: the second components of t‐SNE projections
t‐SNE‐MCP‐k: t‐SNE of KC‐data
t‐SNE‐MCP‐O: the optimal t‐SNE‐MCP‐k map for clusters of MCP
t‐SNE‐MCP: t‐SNE of (*n* − 1)‐data
t‐SNE‐N: t‐SNE of the normalized points
t‐SNE: *t*‐statistic stochastic neighbor embedding

Clustering analysis is used to search for patterns and group genes into expression clusters that provide additional insight into the biological function and relevance of genes that show different expressions 1. The most popular clustering algorithms include hierarchical clustering 2, 3, *k* means clustering 4, and self‐organizing maps (SOMs) 5. However, clustering analysis cannot reveal underlying global patterns in the data, or relationships between the clusters found. To complement clustering analysis, dimension reduction techniques map the high‐dimensional points onto a 2D or 3D visualization space that is displayed graphically as a scatter plot, which provides a humanly interpretable visualization of the data set. A commonly used method for this purpose is principal component analysis (PCA). But for complex gene expression data sets, PCA typically gives poor visualizations 6, 7. Because of these limitations, non‐linear dimension reduction methods have been used to preserve local structure in the data, such as *t*‐statistic stochastic neighbor embedding (t‐SNE) 7, 8, 9, 10. t‐SNE has been successful in complementing clusters of Euclidean distance, but it is usually inefficient for displaying clusters of Pearson's correlation coefficient (PCC). For instance, for any reference data set in this paper, t‐SNE gives poor visualizations for the clusters that are generated by PCC.

Here, we use Pearson's correlation coefficient of multiple‐cumulative probabilities (PCC‐MCP) as a measure to define similarity of genes, where MCPs are composed by *n* cumulative probabilities of genes, and *n* is the dimension of gene points. These cumulative probabilities of each gene are generated from *n* permutations of the normalized points. For permutations of a normalized point, they have the same elements as the normalized points, but their element orders are different. Compared to the normalized points, MCPs are able to weaken the curve shape difference of genes with similar expression behavior. For instance, these genes can be seen as similar expression behavior that their elements are relatively equivalent, but their shape curves may have differences. Moreover, MCPs enlarge the element discrepancy of dissimilar genes. To evaluate the reliability of PCC‐MCP, we apply it with icc‐cluster to group a simulated data set and four experimental expression data sets. When PCC‐MCP with icc‐cluster applies to these data sets, it produces clusters of more statistical relevance than those generated by some other popular clustering methods. This superior performance of PCC‐MCP partially confirms the validity of MCPs. Moreover, icc‐cluster with PCC‐MCP has great ability to remove the effect of the clustering numbers. In fact, even if clustering number is relative large or small compared to the optimal one, icc‐cluster is able to attain tight and stable clusters.

Here, we firstly construct KC‐data sets, where KC‐data is composed of the first to *k*‐th principal components of MCPs, these components of MCPs are generated from PCA, *k* is less than *n*, and *n* is the dimension of the genes. Then, t‐SNE‐MCP‐k map is generated from t‐SNE of KC‐data, where t‐SNE‐MCP‐k is 2D projections of the KC‐data. And then, t‐SNE‐MCP‐O map is selected from these t‐SNE‐MCP‐k projections by the average silhouette value of clustering results. That is, t‐SNE‐MCP‐O is such t‐SNEMCP‐k map that has the most clear boundaries for 2D projections of clustering results. To evaluate the reliability of t‐SNE‐MCP‐O, we use it to display clusters of a simulated data set and four experimental expression data sets, where clusters are generated from PCC‐MCP. Results show that t‐SNE‐MCP‐O gives clearer projecting boundaries for these clusters than commonly used dimension‐reduction techniques. Furthermore, to readily see which nearby 2D points are truly similar, we also construct gene neighbor maps by t‐SNE‐MCP‐(*n* – 1). Results show that t‐SNE‐MCP‐(*n* – 1) makes the relationships between clusters easy to visualize and understand.

In this study, icc‐cluster with PCC‐MCP is firstly applied to yeast metabolic cycle data 11, mouse retina data 12, human embryo data 13 and K562 cell line data 14, and then these clustering results are overlayed onto t‐SNE‐MCP‐O maps, which makes the identification of gene relationships easy and intuitive.

## Materials and methods

### Data set 1

The simulation data set consisted of 2000 members at five time points, which were generated from normal distributions. These 2000 members belonged to four groups, A, B, C and D, according to the models they were generated from, and each group consisted of 500 members. The members of groups that were generated from the different normal distributions are shown in Table 1.

**Table 1:** List of simulated data

Time points	Group A	Group B	Group C	Group D
1	*N*(10,2)	*N*(10,2)	*N*(100,10)	*N*(10,2)
2	*N*(10,2)	*N*(0.5,0.1)	*N*(60,6)	*N*(30,3)
3	*N*(10,2)	*N*(0.5,0.1)	*N*(60,6)	*N*(10,2)
4	*N*(10,2)	*N*(0.5,0.1)	*N*(60,6)	*N*(60,6)
5	*N*(10,2)	*N*(30,3)	*N*(100,10)	*N*(10,2)

### Data set 2

This data set consisted of yeast metabolic cycle data: NCBI GEO accession number GSE3431. It described the transcriptional changes in the metabolic cycle of budding yeast, *Saccharomyces cerevisiae* 11. In this experiment, gene expression behaved in a periodic manner, comprising a non‐respiratory phase followed by a respiratory phase. The transcriptome was assayed every 25 min over three consecutive cycles, resulting in 36 samples (T1–T36). These were profiled using Affymetrix YG_S98 oligonucleotide arrays (Affymetrix Inc., Santa Clara, CA, USA). Probes that had at least three present calls generated by Affymetrix gene chip software were classified as expressed and the data normalized using genespring v7 per‐chip normalization. Using a periodicity algorithm described in the original paper, the authors classified 3552 genes as periodic, corresponding to 3656 probe sets. From these 3656 probe sets, 2913 probes, expression values were greater than 5 in at least one of 36 samples selected. These 2913 probes were summarized in Data S2.

### Data set 3

The raw mouse retinal data consisted of 10 SAGE libraries (38 818 unique tags with tag counts ≥ 2) from developing retina taken at 2‐day intervals. The samples ranged from embryonic, to postnatal, to adult. Among the 38 818 tags, 1467 tags that had counts ≥ 20 in at least one of the 10 libraries were selected 12. The purpose of this selection was to exclude the genes with uniform low expression. The counts of each tag in a SAGE library were Poisson distributed. These Poisson distributions were independent of each other across different tags and libraries 12, 15. These 1467 tags are summarized in Data S3.

### Data set 4

This data set consisted of human embryo data: NCBI GEO accession number GSE18887. The resulting matrix contained expression measurements for 5441 transcripts across 18 samples, denoted as the human organogenesis expression matrix 13 (Carnegie stages 9–14, S9–S14). A total of 5441 probe sets were identified as differentially expressed using extraction of differential gene expression (EDGE)‐based methodology. Initially, Fang *et al*. used SOM‐SVD (SOM combined with singular value decomposition) to identify co‐expressed genes of human embryo data 13, which identified six clusters. From their analysis, they extracted 2148 differentially expressed probe sets. We used this set of 2148 probe sets for our analysis. These 2148 probes are summarized in Data S4.

### Data set 5

NCBI GEO accession number GSE12736. Time course microarray data were obtained at seven independent time points. Duplicate experiments were performed for each time point. Selecting genes with a significant detection *P*‐value produced 14 000 probes out of a total of 23 920 probes. Quantile normalization was carried out for each data set at seven time points using the average expression value. It was reasoned that significant genes should show over two‐fold induction at least at one time point with respect to the control sample (*t* = 0; before phorbol 12‐myristate 13‐acetate treatment), and 1779 probes satisfying this requirement had been determined 14, 16. These 1779 probes are summarized in Data S5.

### MCPs of genes

Here, *X*_*i*_ = {*x*_*i*1_, *x*_*i*2_, ···, *x*_*in*_} represents the *i*‐th gene, and *x*_*ij*_ represents the expression level of the *j*‐th time points. Here, we sketch MCP of *X*_*i*_ as follows.

(a) *X*_*i*_ is normalized into *Y*_*i*_, where

$$Y_{i}=\left\{y_{i1},y_{i2},\cdots ,y_{in}\right\},y_{it}=\frac{x_{it}-\min\left(\min_{1\leq t\leq n}\left(x_{it}\right),0\right)}{\sum_{l=1}^{n}\left(x_{il}-\min\left(\min_{1\leq t\leq n}\left(x_{it}\right),0\right)\right)},\;t=1,2,\cdots ,n.$$

For genes, expression levels may be negative at some time points, such as the genes of data set 5. Here, we deal with these genes by a translational transformation. That is, *x*_*it*_ is substituted by $x_{it}-\min\left(\min_{1\leq t\leq n}\left(x_{it}\right),0\right)$. In fact, if all expression levels of *X*_*i*_ are non‐negative, $x_{it}-\min\left(\min_{1\leq t\leq n}\left(x_{it}\right),0\right)$ is the same as *x*_*it*_. Moreover, *Z*_*is*_ is constructed, where *Z*_*is*_ is the *s*‐th permutation of *Y*_*i*_, and

$$Z_{is}=\left\{y_{is},y_{i(s+1)},\cdots ,y_{in},y_{i1},y_{i2},\cdots ,y_{i(s-1)}\right\},s=1,2,\cdots ,n.$$

Based on *Z*_*is*_, *T*_*is*_ is constructed, where

(1) $$\begin{array}{cc} T_{is}= & \{y_{is}/2,y_{is}+y_{i(s+1)}/2,\cdots ,\sum_{l=s}^{n-1}y_{il}+y_{in}/2,\sum_{l=s}^{n}y_{il}+y_{i1}/2,\\ & \sum_{l=s}^{n}y_{il}+y_{i1}+y_{i2}/2,\cdots ,\sum_{l=s}^{n}y_{il}+\sum_{l=1}^{s-2}y_{il}+y_{i(s-1)}/2\} \end{array}.$$

That is, *T*_*is*_ is the modified cumulative probability of *Z*_*is*_.

(b) Based on *T*_*is*_, *T*_*i*_ is constructed, where *T*_*i*_ is an *n* ^2^‐dimensional vector, and

$$T_{i}=\left\{T_{i1},T_{i2},\cdots ,T_{in}\right\}.$$

Here, *T*_*i*_ is considered to be the MCP of *X*_*i*_.

**Figure 1:**
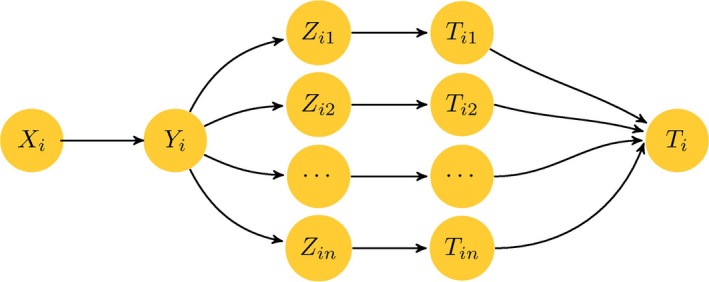
Flowchart of the multiple‐cumulative probabilities.

To clearly understand MCPs, we used a flowchart to show their construction process (Fig. 1). Moreover, we define Euclidean distance and PCC between *T*_*i*_ and *T*_*j*_ as the Euclidean distance of multiple‐cumulative probabilities (ED‐MCP) and PCC‐MCP of *X*_*i*_ and *X*_*j*_, respectively.

### icc‐cluster algorithm

(a) Choose *X*_*j*1_ and *X*_*j*2_ as the first and second cluster centers, where

$$d_{j1,j2}=\max_{1\leq i<j\leq m}\left\{d_{i,j}\right\},$$

*m* is the gene number of the data set. If *X*_*js*_ satisfies

$$\begin{array}{cc} \min & \left\{d_{js,j1},d_{js,j2},\cdots ,d_{js,j(s-1)}\right\}\\ & =\max_{1\leq i\leq m,i\neq j1,\cdots ,j(s-1)}\min\left\{d_{i,j1},d_{i,j2},\cdots ,d_{i,j(s-1)}\right\} \end{array},$$

it is chosen as the *s* (*s* = 4, 5, ···, *k*)‐th cluster center.

(b) *X*_*i*_ belongs to the *l*‐th cluster (1 ≤ *l* ≤ *k*) if it satisfies

$$d_{i,jl}=\min\left\{d_{i,j1},d_{i,j2},\cdots ,d_{i,jk}\right\}.$$

(c) Assuming *X*_*l*1_, *X*_*l*2_, ···, *X*_*lq*_ in the *l*‐th cluster, *X*_*li*_ is chosen as a new center if it satisfies

$$\sum_{j=1}^{q}d_{li,\;lj}=\min_{1\leq m\leq q}\sum_{j=1}^{q}d_{lm,\;lj}.$$

(d) Repeat step 2 and step 3 until the assignment does not change.

For the icc‐cluster algorithm, when PCC is used to measure the similarity of genes,

$$d_{i,j}=1-\varphi_{i,j},$$

where $\varphi_{i,j}$ is PCC between *X*_*i*_ and *X*_*j*_ (or *T*_*i*_ and *T*_*j*_).

### The t‐SNE‐MCP‐O and t‐SNE‐MCP‐k maps

The formal description of the t‐SNE algorithm can be found in Ref. 9. But for the high‐dimensional expression points of genes, it is difficult for us to obtain t‐SNE projections of their MCPs. To answer the challenge of the high dimensional MCPs, we use t‐SNE‐MCP‐O to display clusters of PCC‐MCP, where t‐SNE‐MCP‐O selects from t‐SNE‐MCP‐k (*k* = 2, 3, ···, *n* − 1) maps, t‐SNE‐MCP‐k is t‐SNE of KC‐data, points of KC‐data are composed of the first to *k*‐th principal components of *T*_*i*_, components of *T*_*i*_ were generated from PCA. In fact, for PCA of MCPs, only their first to (*n* − 1)‐th principal components are not zero, and we abbreviate t‐SNE‐MCP‐(*n* − 1) as t‐SNE‐MCP.

Here, for clusters of PCC‐MCP, we use the average silhouette value of t‐SNE‐MCP‐k to select their t‐SNE‐MCP‐O. The average silhouette value of t‐SNE‐MCP‐k is defined as

$$S_{k}=\frac{1}{m}\sum_{i=1}^{m}\frac{\left(b_{i}-a_{i}\right)}{\max(a_{i},b_{i})},$$

where *a*_*i*_ is the average Euclidean distance from *U*_i_ to the other points in the same cluster as *U*_i_, *b*_*i*_ is the minimum average distance from *U*_*i*_ to points in a different cluster, minimized over clusters, *U*_*i*_ is the t‐SNE projection of *P*_*i*_, *P*_*i*_ is the *i*‐th point of KC‐data, and *m* is the gene number of the data 17. Moreover, if *T*_*i*_ belongs to the *a*‐th cluster, cluster membership of *U*_*i*_ is *a* also. For these *S*_*k*_ of clusters that generate from PCC‐MCP, if *S*_*k*_ is the largest, t‐SNEMCP‐k is selected as the t‐SNE‐MCP‐O map of the clusters. For convenience, we use t‐SNE‐MCP‐1 to denote t‐SNE of MCPs.

## Results

Here, all clustering results were generated from the normalized points, and PCC‐MCP, PCC, ED‐MCP, Euclidean distance, TransChisq and PoissonC were chosen as measures of genes. Moreover, clustering number of data sets mainly came from the corresponding references. In detail, data set 2 had been divided into eight clusters by Euclidean distance 7, data set 3 into 25 clusters by PoissonC and TransChisq 12, 15, data set 4 into six and 10 clusters by Euclidean distance 7, and data set 5 into eight clusters by Euclidean distance 14.

### Comparison of PCC‐MCP and PCC

**Figure 2:**
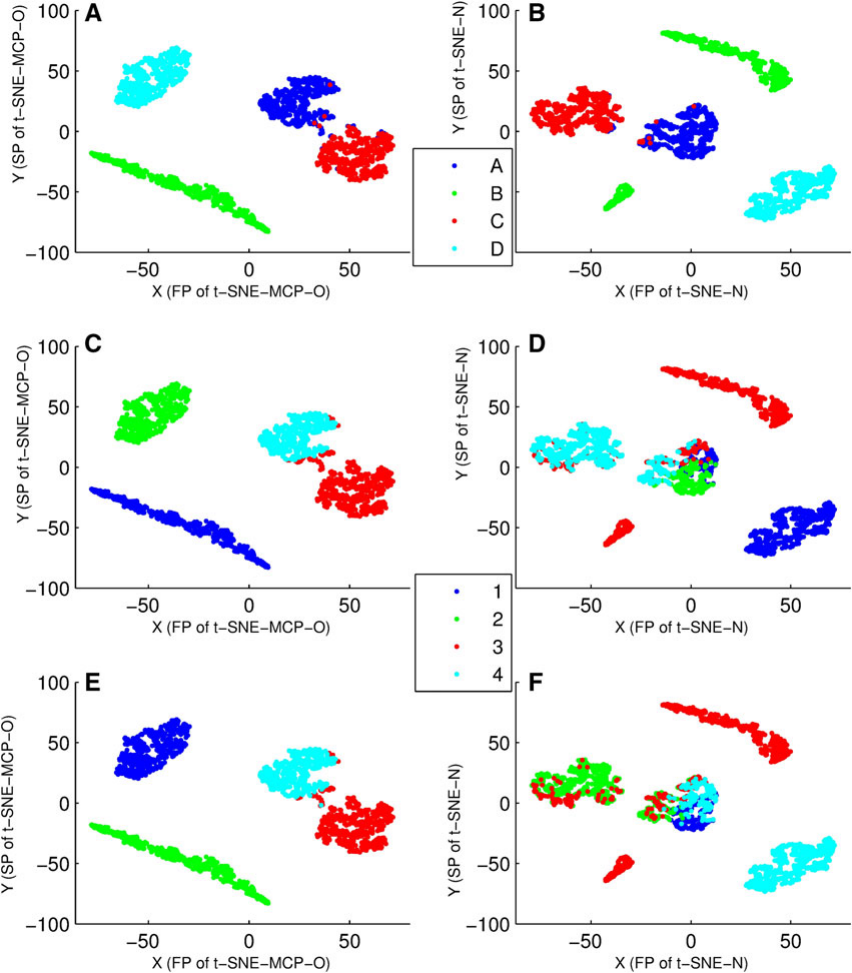
Overlay of clusters of data set 1 onto 2D maps, where data points were colored according to cluster membership. (A) Overlay of four populations onto t‐SNE‐MCP‐O map. (B) Overlay of four populations onto t‐SNE‐N map. (C) Overlay of four clusters onto t‐SNE‐MCP‐O map, where clusters were generated from icc‐cluster with PCC‐MCP. (D) Overlay of four clusters onto t‐SNE‐N map, where clusters were generated from icc‐cluster with PCC. (E) Overlay of four clusters onto t‐SNE‐MCP‐O map, where clusters were generated from *k* means with PCC‐MCP. (F) Overlay of four clusters onto t‐SNE‐N map, where clusters were generated from *k* means with PCC.

Here, data set 1 was firstly displayed on t‐SNE‐MCP‐O and t‐SNE‐N maps according to population membership of points (Fig. 2A,B), where t‐SNE‐MCP‐O of data set 1 was t‐SNE‐MCP‐3, and population membership of points was summarized in Table 1. From Fig. 2A,B, t‐SNE‐MCP‐O and t‐SNE‐N correctly separated members of different groups, and only a few members of group A were assigned to group C, where members of group A and group C (Table 1) were marked by the blue and red colors in Fig. 2A,B, respectively. Moreover, data set 1 was divided into four clusters by PCC‐MCP and PCC, where icc‐cluster and *k* means were used simultaneously. Then, these clustering results were displayed on t‐SNE‐MCP‐O and t‐SNE‐N maps (Fig. 2C–F).

From Fig. 2A,C,E, for clusters of PCC‐MCP, icc‐cluster and *k* means correctly assigned members of group B, C and D into three different clusters, and only a few members of group A were assigned to the cluster that contained all the points of group C. But for clusters of PCC, icc‐cluster and *k* means assigned members of group A to four different clusters, and assigned members of group C to three different clusters (Fig. 2B,D,F). For members of group A, their elements of five time points came from *N*(10,2) simultaneously, so their elements were relatively equivalent, but their shape curves had significant differences. The poor performance of PCC may be due to the fact that PCC only compared the shape of the curves. For instance, PCC between *a*1 = (8.08, 12.36, 9.86, 10.63, 11.14) and *a*4 = (12.39, 9.91, 9.54, 9.35, 9.41) was only −0.74, while PCC between *a*1 and *d*384 = (6.49, 37.18, 9.12, 49.39, 13.12) was 0.594, where *a*1 and *a*2 came from group A: and *d*384 came from group D. However, PCC‐MCP between *a*1 and *a*4 was 0.993, while PCC‐MCP between *a*1 and *d*384 was 0.95. That is, the defect of PCC was removed by MCPs.

### The statistical reliability of icc‐cluster with PCC‐MCP

The average silhouette value was a quantitative way to evaluate the clustering solutions 17, and we used it to demonstrate the reliability of icc‐cluster with PCC‐MCP. Here, we applied ED‐MCP, PCC‐MCP, PCC and Euclidean distance to four experimental data sets. For comparison, icc‐cluster and *k* means were used simultaneously, and the average silhouette values of these clustering results were summarized in Table 2. For all data sets, Table 2 showed that their largest average silhouette value came from icc‐cluster, and three of these largest average silhouette values were generated by PCC‐MCP, where the largest average silhouette value of each data set was marked by an asterisk in Table 2. Moreover, for clustering results of any data, the average silhouette value of ED‐MCP was far less than PCC‐MCP. That is, clusters of PCC‐MCP were better separated from neighboring clusters than ED‐MCP.

**Table 2:** The average silhouette values of different measures. Number: clustering number. For each data set, the largest average silhouette value was marked by an asterisk

Data	Algorithm	Number	ED‐MCP	PCC‐MCP	Euclidean distance	PCC
2	icc‐cluster	3	0.24905	0.40003	0.48845*	0.47263
2	*k* means	3	0.25236	0.40542	0.26024	0.47327
3	icc‐cluster	25	0.18948	0.31151*	0.13419	0.22494
3	*k* means	25	0.18638	0.30046	0.15225	0.23674
4	icc‐cluster	10	0.32985	0.53427*	0.27882	0.20489
4	*k* means	10	0.14063	0.31636	0.16123	0.11007
5	icc‐cluster	12	0.26081	0.41184*	0.23601	0.30403
5	*k* means	12	0.16611	0.2457	0.17662	0.38694

### The biological reliability of icc‐cluster with PCC‐MCP

Here, 34 ‘cell‐specific’ tags of data set 3 were used to test the biological reliability of icc‐cluster with PCC‐MCP, where these 34 tags are summarized in Data S3. Moreover, these ‘cell‐specific’ tags showed the most dynamic and cell‐specific expression in the mouse neonatal retina (developmental stages *P*_0_–*P*_6_) 12. Data set 3 had been grouped into 25 clusters by TransChisq and PoissonC measure 12, 15, respectively. Moreover, these 34 cell‐specific genes were used to demonstrate that TransChisq and PoissonC measures were more efficient for analyzing SAGE data than PCC and Euclidean distance. For comparison, we used icc‐cluster with PCC‐MCP to group these 1467 tags into 25 clusters also. Then, for each of the different algorithms, the five most dynamic clusters that contained ‘cell‐specific’ tags were selected. The comparison statistics of 34 ‘cell‐specific’ tags were summarized in Table 3. In Table 3, icc‐cluster generated clusters that were most enriched for these 34 cell‐specific genes. That is, PCC‐MCP was appropriate and reliable for analyzing SAGE data also.

**Table 3:** Statistics of 34 ‘specific’ genes of data set 3. The second column showed the numbers of cell‐specific genes in a cluster; total, the total number of cluster members; sensitivity, the number of cell‐specific genes/34; precision, the number of cell‐specific genes/total number of cluster members. The top five clusters that contained the 34 cell‐specific genes are listed. The numbers in bold were the highest percentage for sensitivity and precision for that method

	Cell‐specific genes	Total	Sensitivity (%)	Precision (%)
PCC‐MCP	11	41	**32.3**	**26.8**
	1	5	2.8	20
	2	14	5.9	14.3
	5	38	14.7	13.2
	3	27	8.8	11.1
TransChisq	9	41	**26.5**	**22.0**
	1	12	2.9	8.3
	3	39	8.8	7.7
	1	24	2.9	4.2
	3	74	8.8	4.1
PoissonC	10	47	**29.4**	**21.3**
	4	22	11.8	18.2
	3	27	8.8	11.1
	1	11	2.9	9.1
	1	12	2.9	8.3

### The features of the MCPs

**Figure 3:**
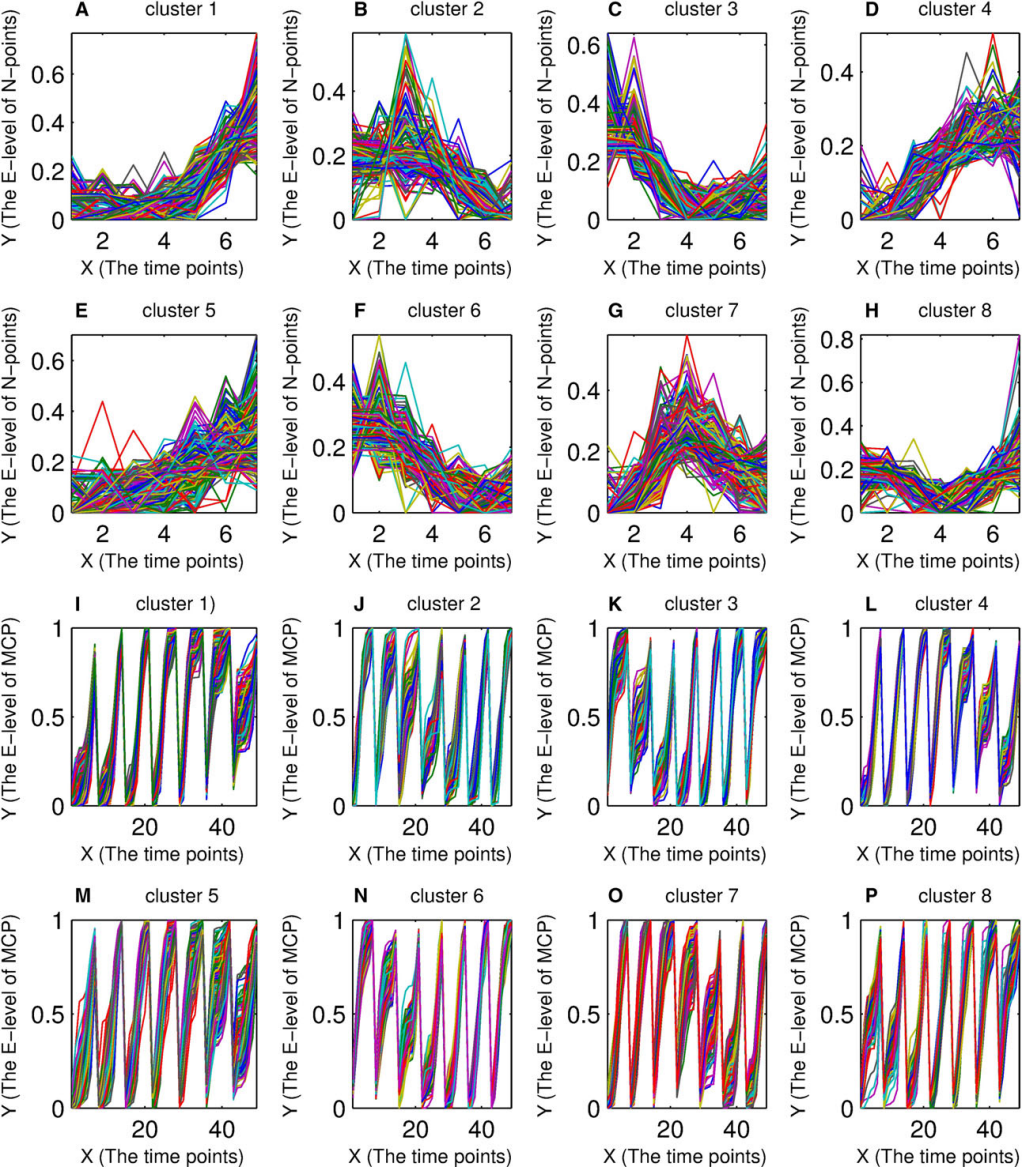
The profile plots of the normalized points and MCPs. The *X*‐axis represented the different time points. The *Y*‐axis represented the expression level, where E‐level is the abbreviation of expression level. (A–H) The profiles of normalized plots of eight clusters; (I–P) The profile of the multiple‐cumulative probabilities of eight clusters.

By PCC‐MCP with icc‐cluster, data set 5 was firstly divided into eight clusters. Then, these clusters were selected to explore the feature of MCPs. For MCPs and normalized points of each cluster, their curve shapes were shown in Fig. 3. For the curve shapes of MCPs, Fig. 3 showed that they were almost the same in the same clusters, but had significant differences in the different clusters. That is, MCPs weakened the curve shape difference of the genes with similar expression behavior, but enlarged the element discrepancy of dissimilar genes. Importantly, for each of clusters that were generated from PCC‐MCP, the curve shapes of their normalized points had no significant difference (Fig. 3A–H).

### The features of icc‐cluster

Here, we used data set 2 to validate that icc‐cluster had great ability to remove the effect of clustering numbers, where data set 2 was divided into three and five clusters by PCC‐MCP. The comparison statistics of super‐clusters and sub‐clusters are summarized in Table 4. For each sub‐cluster of icc‐cluster, Table 4 showed that it contained 85% genes that came from the same super‐cluster at least. However, for two sub‐clusters of *k* means, their genes came from the same super‐cluster that were < 70%.

**Table 4:** Statistics of three superclusters and five subclusters of data set 2. The second column shows the number of subclusters. The numbers in the third, fourth and fifth column were the number of genes in subclusters that came from the three superclusters. Total was the total number of subcluster members. The sensitivity was the bold numbers/total number of subcluster members

	No.	1	2	3	Total	Sensitivity (%)
icc‐cluster	1	**16**	0	0	16	100.00
	2	**257**	0	0	257	100.00
	3	0	0	**1458**	1458	100.00
	4	14	**996**	106	1116	89.25
	5	**65**	11	0	76	85.53
*k* means	1	**431**	4	220	655	65.80
	2	0	**496**	0	496	100.00
	3	0	0	271	271	100.00
	4	**828**	393	0	1221	67.81
	5	**293**	0	17	310	94.52

**Figure 4:**
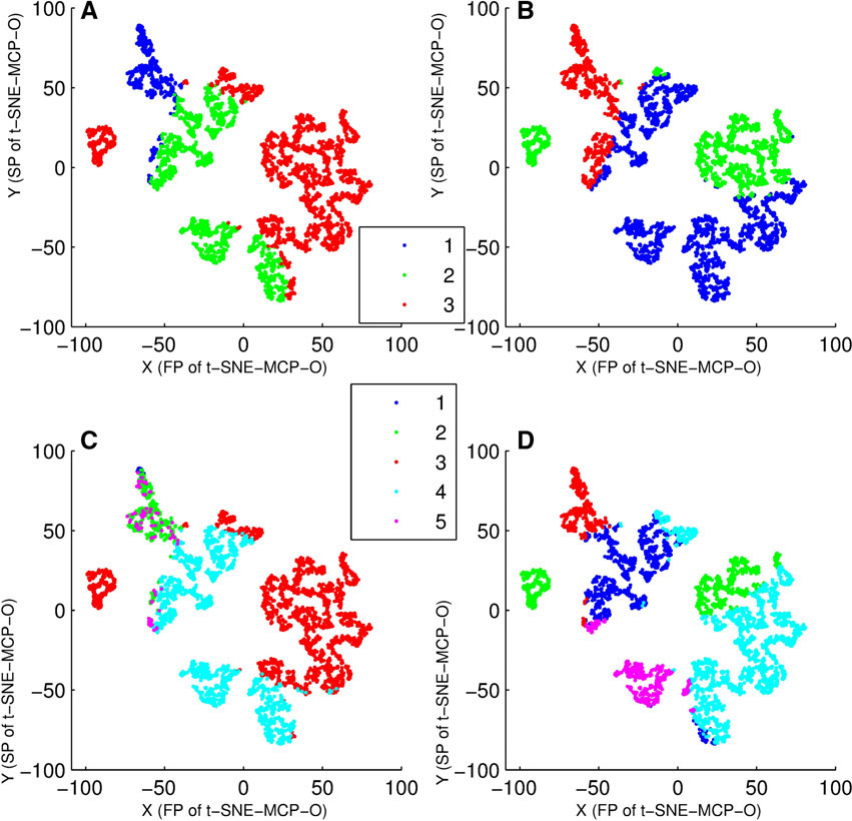
Overlay of clusters of data set 2 onto t‐SNE‐MCP‐O (t‐SNE‐MCP‐2) maps, where data points were colored according to cluster membership. (A) Overlay of three superclusters of icc‐cluster onto t‐SNE‐MCP‐O map. (B) Overlay of three superclusters of *k* means onto t‐SNE‐MCP‐O map. (C) Overlay of five subclusters of of icc‐cluster onto t‐SNE‐MCP‐O map. (D) Overlay of five subclusters of *k* means onto t‐SNE‐MCP‐O map.

Moreover, we used t‐SNE‐MCP‐O (t‐SNE‐MCP‐2) maps to display the features of icc‐cluster (Fig. 4). For sub‐clusters of icc‐cluster, Fig. 4A,C showed that they mainly came from the same super‐clusters. From Fig. 4B,D, clustering results of *k* means were significantly affected by clustering numbers.

### The consistency between clusters of PCC‐MCP and t‐SNE‐MCP

**Figure 5:**
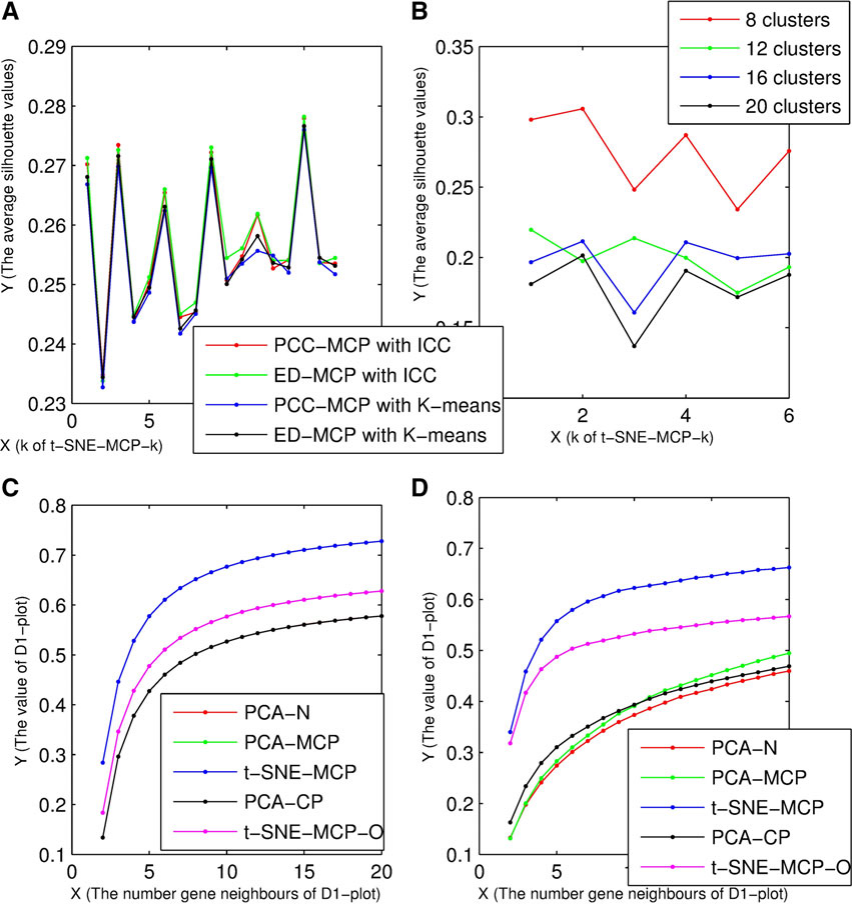
The average silhouette values of t‐SNE‐MCP‐k and *D*_1_‐plot of data set 4 and 5. (A) The average silhouette values of t‐SNE‐MCP‐k maps of data set 4, where clustering results of PCC‐MCP with icc‐cluster, ED‐MCP with icc‐cluster, PCC‐MCP with *k* means and ED‐MCP with *k* means were shown by red, green, blue and gray line, respectively. (B) The average silhouette values of all t‐SNE‐MCP‐k maps of data set 5, where 8, 12, 16 and 20 clusters were shown by red, green, blue and gray line, respectively. (C) *D*_1_‐plot of data set 4, where *D*_1_‐plot of t‐SNE‐MCP, t‐SNE‐MCP‐O, PCA‐MCP, PCA‐CP and PCA‐N were shown by blue, pink, green, gray and red line, respectively. (D) *D*_1_‐plot of data set 5, where *D*_1_‐plot of t‐SNE‐MCP, t‐SNE‐MCP‐O, PCA‐MCP, PCA‐CP and PCA‐N were shown by blue, pink, green, gray and red line, respectively.

For a visualization technique, if it was able to project genes of the same clusters together, and project neighbor clusters in adjacent regions, we considered that it was consistent with clustering results. In general, we assessed the consistency by eye, which was an intuitive feeling only. Here, the average silhouette value was used to quantify the consistency, where we focused on data set 4 and 5. Firstly, data set 4 was divided into 10 clusters by PCC‐MCP (or ED‐MCP) with icc‐cluster (or *k* means). For any clustering result of data set 4, the average silhouette values of its t‐SNE‐MCP‐k maps were shown in Fig. 5A. From Fig. 5A, for t‐SNE‐MCP, the average silhouette values of any clustering result were less than t‐SNE‐MCP‐15. That is, t‐SNE‐MCP‐15 was t‐SNE‐MCP‐O of data set 4, and t‐SNEMCP‐1 did not optimally display clusters of MCPs. Secondly, data set 5 was divided into 8, 12, 16 and 20 clusters by PCC‐MCP with icc‐cluster. For any clustering result of data set 4, the average silhouette values of its t‐SNE‐MCP‐k maps were shown in Fig. 5B. From Fig. 5B, for 8, 16 and 20 clusters, t‐SNE‐MCP‐2 was their t‐SNE‐MCP‐O. But for 12 clusters, the average silhouette value t‐SNE‐MCP‐1 was the largest. That is, for different clustering number, their t‐SNE‐MCP‐O maps were not consistent.

### The local validity of t‐SNE‐MCP

We considered that a dimension‐reduction technique was locally valid if the *i*‐th closest neighbour of a point was its *j*‐th closest neighbour in 2D space, where *i*,* j* and |*i* − *j*| were the relative small numbers, point neighbours were located by any measure, while projection neighbours were located by Euclidean distance 6. Moreover, the local validity of dimension reduction techniques could be quantified by *D*_1_‐plot 6.

Here, data set 4 and 5 were used to assess the local validity of t‐SNE‐MCP, t‐SNE‐MCP‐O, PCA‐MCP, PCA‐CP and PCA‐N, where we named the PCA of the normalized points and cumulative probabilities as PCA‐N and PCA‐CP, respectively. Moreover, when gene neighbours were defined by PCC of MCPs, the normalized points and cumulative probabilities, their neighboring maps corresponded to t‐SNE‐MCP (t‐SNE‐MCP‐O and PCA‐MCP), PCA‐CP and PCA‐N, respectively. Moreover, *D*_1_‐plots of data set 4 and 5 are shown in Fig. 5C and D, respectively. From Fig. 5C,D, the local validity of t‐SNE‐MCP was far more than others. That is, t‐SNE‐MCP better preserved local proximities of genes compared with other dimension‐reduction techniques.

### Comparison of t‐SNE‐MCP and t‐SNE‐MCP‐O

**Figure 6:**
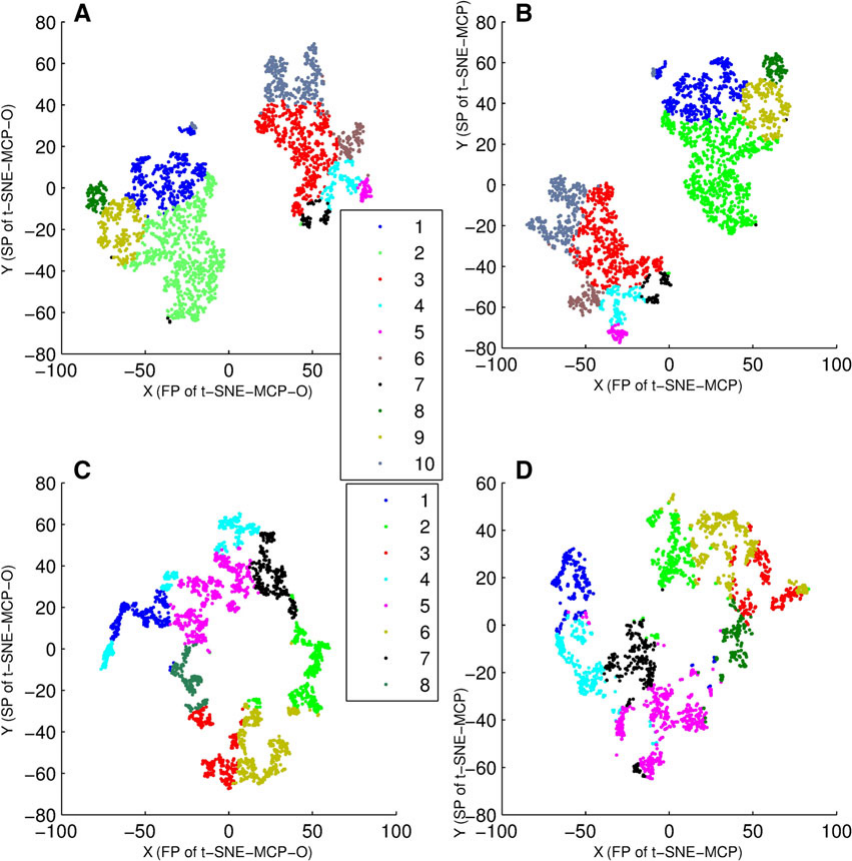
Overlay of clusters of data set 4 and 5 onto t‐SNE maps, where clusters were generated from PCC‐MCP with icc‐cluster, and data points were colored according to cluster membership. (A) Overlay of 10 clusters of data set 4 onto t‐SNE‐MCP‐O (t‐SNE‐MCP‐15) map. (B) Overlay of 10 clusters of data set 4 onto t‐SNE‐MCP map. (C) Overlay of eight clusters of data set 5 onto t‐SNE‐MCP‐O (t‐SNE‐MCP‐2) map. (D) Overlay of eight clusters of data set 5 onto t‐SNE‐MCP map.

By PCC‐MCP with icc‐cluster, data sets 4 and 5 were firstly divided into 10 and 8 clusters, respectively. Then, these clustering results were displayed on t‐SNE‐MCP and t‐SNE‐MCP‐O maps (Fig. 6). As shown in Fig. 6A,B, t‐SNE‐MCP‐O and t‐SNE‐MCP gave good projections for clusters of data set 4, where t‐SNE‐MCP‐O was t‐SNE‐MCP‐15. For clusters of data set 5, t‐SNE‐MCP‐O (t‐SNE‐MCP‐2) gave clearly projecting boundaries (Fig. 6C), while their t‐SNE‐MCP map showed slight intermixing (Fig. 6D). In fact, for data sets 2 and 3, t‐SNE‐MCP maps were not the optimal projections for clusters of PCC‐MCP also.

### Comparison of t‐SNE‐MCP‐O, t‐SNE‐N and t‐SNE‐C

**Figure 7:**
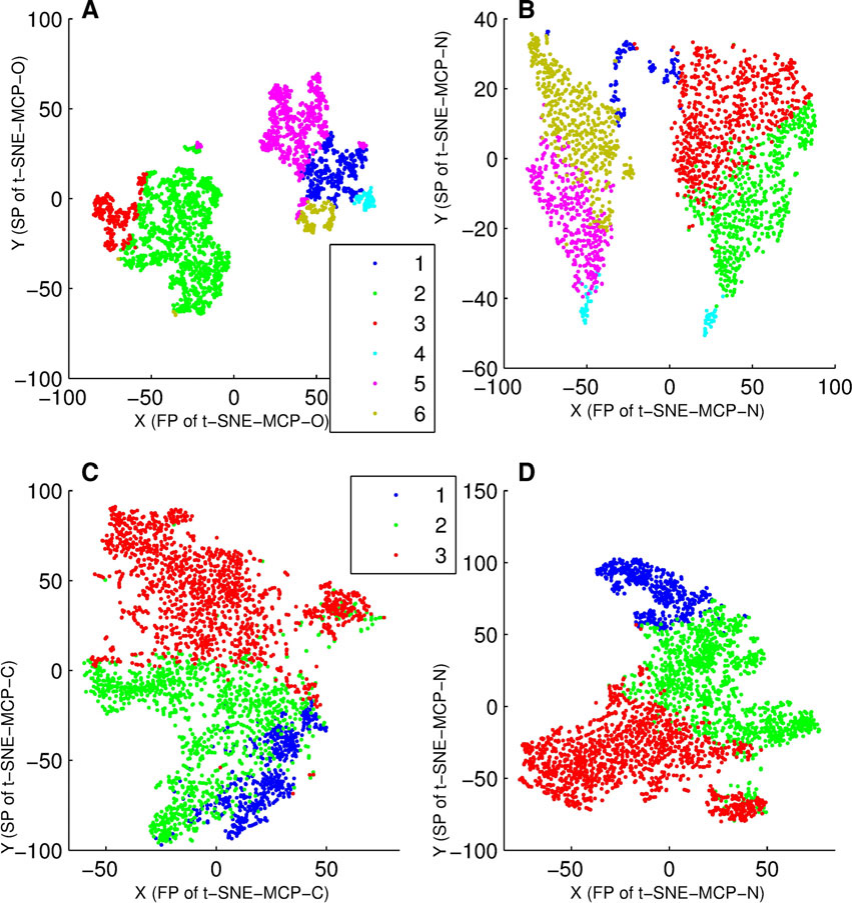
Overlay of clusters of data set 4 and 2 onto t‐SNE‐MCP‐O, t‐SNE‐N and t‐SNE‐C maps, where data points were colored according to cluster membership. (A) Overlay of six clusters of data set 4 onto t‐SNE‐MCP‐O (t‐SNE‐MCP‐15) map, where clusters were generated from PCC‐MCP with *k* means. (B) Overlay of six clusters of data set 4 onto t‐SNE‐N map, where clusters were generated from PCC with *k* means. (C) Overlay of three clusters of data set 2 onto t‐SNE‐C map, where clusters were generated from PCC with *k* means. (D) Overlay of three clusters of data set 2 onto t‐SNE‐N map, where clusters were generated from PCC with *k* means.

Here, data set 4 was divided into six clusters by PCC‐MCP (or PCC) with *k* means, and data set 2 into three clusters by PCC with *k* means. Moreover, these clustering results were displayed on t‐SNE‐MCP‐O, t‐SNE‐N and t‐SNE‐C maps (Fig. 7), where t‐SNE‐C was t‐SNE of the centered gene points 7. From Fig. 7, only t‐SNE‐MCP‐O gave clearly projecting boundaries for clusters of PCC‐MCP (Fig. 7A), while t‐SNE‐N and t‐SNE‐C maps had significant intermixing for any clustering result of PCC (Fig. 7B,C,D).

### Comparison of ED‐MCP and Euclidean distance

**Figure 8:**
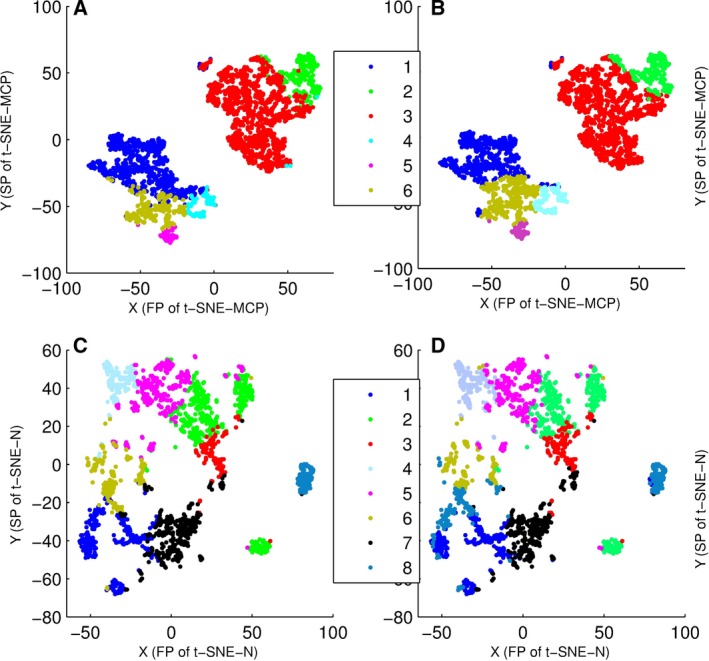
Overlay of clusters of data set 4 and 5 onto t‐SNE‐MCP and t‐SNE‐N maps, where data points were colored according to cluster membership. (A) Overlay of six clusters of data set 4 onto t‐SNE‐MCP map, where clusters were generated from ED‐N with *k* means. (B) Overlay of six clusters of data set 4 onto t‐SNE‐MCP map, where clusters were generated from ED‐MCP with *k* means. (C) Overlay of eight clusters of data set 5 onto t‐SNE‐N map, where clusters were generated from ED‐N with *k* means. (D) Overlay of eight clusters of data set 5 onto t‐SNE‐N map, where clusters were generated from ED‐MCP with *k* means.

By ED‐MCP (or ED‐N) with *k* means, data set 4 was divided into six clusters, and data set 5 into eight clusters, where ED‐N was Euclidean distance of the normalized gene points. Moreover, clustering results of data set 4 were displayed on t‐SNE‐MCP maps (Fig. 8A,B), and clustering results of data set 5 on t‐SNE‐N maps (Fig. 8C,D). From Fig. 8, only t‐SNE‐MCP gave clearly projecting boundaries for clusters of ED‐MCP (Fig. 8B), and t‐SNE‐N maps had slight intermixing. Importantly, Fig. 8 showed that clustering results of ED‐MCP and ED‐N had no significant difference. That is, MCPs retained the difference of the normalized genes.

### The gene neighbors of t‐SNE‐MCP

Hierarchical clustering was commonly used to reveal gene neighbors, for it was much faster and more memory‐efficient 2, 3. However, hierarchical clustering was likely to cause loose gene neighbors. That is, two neighbors that were generated by the hierarchical clustering were not really the nearest and second‐closest neighbors of genes 7.

**Figure 9:**
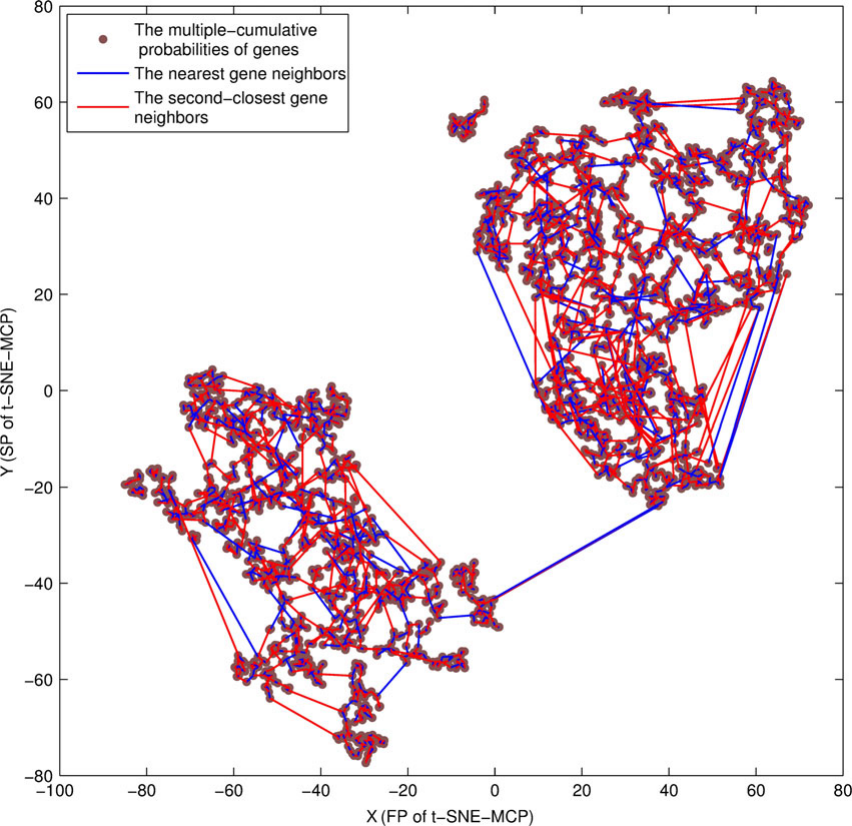
The gene neighbors of data set 4. The nearest and second closest neighbors of genes of data set 4, where the nearest gene neighbors were linked by a blue line, and second‐closest gene neighbors were linked by a red line.

Here, the nearest and second‐closest gene neighbors were generated by PCC‐MCP, where we focused our attention on data set 4. For genes of data set 4, their neighbor maps were shown on a t‐SNE‐MCP map in Fig. 9, where the nearest gene neighbors were linked by a blue line, and second‐closest gene neighbors were linked by a red line. Compared with the hierarchical clustering, the gene neighbor map revealed the pairs of high‐dimensional points that were truly close, and which pairs were in fact distant in 2D space. Moreover, t‐SNE‐MCP maps combined with nearest neighbour maps provided an intuitive means to understand the relationship between clusters and the affiliation of genes with specific clusters.

## Discussion

Although the cumulative probabilities have one‐to‐one mapping with their normalized points, their magnitudes have significant differences, which may distort distance of some similar genes. Moreover, for the different position elements of a normalized point, their superposed opportunity is not consistent in cumulative probability, which can distort the similarity of genes. The defect of cumulative probabilities is removed by MCPs. That is, for MCPs, the magnitudes are the same, and the superposed opportunities of elements in normalized points are consistent.

For high‐throughput data sets, it is difficult for us to obtain t‐SNE projections of the MCPs. Here, for clusters of PCC‐MCP, we use t‐SNE‐MCP‐O to obtain their optimal 2D maps. Moreover, icc‐cluster can relatively rapidly achieve convergence to the optimal solutions. The reason is that icc‐cluster only updates the cluster centers.

## Conclusions

The success of MCPs has two main aspects. One is that MCPs remove the differences of the curve shape of similar expression genes, which makes PCC‐MCP able to robustly measure the similarity of genes. Another is that MCPs enlarge the element divagations of dissimilar genes, which make t‐SNE‐MCP‐O able to clearly display clustering results of PCC‐MCP. We suggest that MCPs can provide new insights applicable to analyzing high‐throughput data. Furthermore, MATLAB implemented PCC‐MCP with icc‐cluster, and from Figs 2–9 are available at Data S1.

## Author contributions

XJ analyzed and discussed the model, and wrote the manuscript. QH and YL performed a portion of the model. ZL supervised the study.

## Supporting information


**Data S1.** A freely available matlab algorithm implemented to obtain clusters from PCC‐MCP with icc‐cluster, and used in Figs 2–9.

**Data S2.** Data set 2 (yeast metabolic cycle data: NCBI GEO accession number GSE3431). Its detail (see Ref. [7]).

**Data S3.** Data set 3 (the raw mouse retinal data consisted of 10 SAGE libraries from developing retina taken at 2‐day intervals). Its detail (see Ref. [12]).

**Data S4.** Data set 4 (human embryo data: NCBI GEO accession number GSE18887). Its detail (see Ref. [7]).

**Data S5.** Data set 5 (NCBI GEO accession number GSE12736). Its detail (see Ref. [14]).
